# Atopic dermatitis and food allergy: when and how to test

**DOI:** 10.1186/2045-7022-1-S1-S24

**Published:** 2011-08-12

**Authors:** Fabienne Rancé

**Affiliations:** 1 Hôpital des Enfants, Allergologie – Pneumologie, Toulouse Cedex, France

## 

Food allergy may provoke flares of atopic dermatitis. The prevalence of food allergy in infants with atopic eczema (AE) may be estimated at 40%. Most of cases of food allergies concern young children (infants aged under one year), and affected by recur eczema under appropriate treatment. Cow's milk, hen' eggs and wheat’ flours are the main food allergens involved in food allergies associated with AE. Food allergies can be identified by clinical history, skin prick test and, IgE specific assays, and the diagnosis confirmed by double-blind, placebo-controlled food challenges (DBPCFC). Oral food challenge is the gold standard to diagnose food allergy in the majority of cases. Nevertheless, method used for oral food challenge should be prolonged on several days according to the late eczematous reactions. Atopy Patch Testing is an approach to diagnose food-induced eczema when the immediate allergic tests are negative. When an allergy is diagnosed, the elimination of an offending food allergen should be added to the medical management of AE.

Food can induce eczematous lesions in young children. Risk factors of food allergy are young age, moderate to severe AE and early onset of AE. Allergic testing recommendations should be done to prove that relationship.
